# The effect of garlic on the lowering of blood pressure in the patients with hypertension: an updated meta-analysis and trial sequential analysis

**DOI:** 10.2478/abm-2025-0016

**Published:** 2025-06-30

**Authors:** Xiao Ma, Hongying Zhang, Jinhai Jia

**Affiliations:** Department of Outpatient, Hebei Medical University, Shijiazhuang, Hebei 050011, China; Graduate School, Hebei Medical University, Shijiazhuang, Hebei 050011, China

**Keywords:** clinical trials, diastolic pressure, garlic extract, meta-analysis, systolic pressure

## Abstract

**Background:**

Garlic extracts have been demonstrated to be effective supplements for reducing blood pressure in hypertensive subjects. However, contradictory observations on the efficacy of garlic extracts have been reported in different studies.

**Objectives:**

To explore the efficacy of garlic, this study aimed to perform a meta-analysis of previously published controlled placebo trials and drew firm conclusions.

**Methods:**

We searched online databases, including Scopus, PubMed, and Science Direct, to obtain relevant articles on the role of garlic in reducing blood pressure in patients with hypertension. The literature search, data extraction, and analysis were performed independently by two researchers. Comprehensive Meta-Analysis Software v4 was used for all the analyses.

**Results:**

Twelve reports, comprising 405 patients treated with garlic derivatives and 333 receiving placebo, were included in this investigation. The results of the meta-analysis revealed a significant decrease in systolic blood pressure (mean difference: −8.121, 95% confidence interval [CI]: −10.95 to −5.28, *P* < 0.0001) and diastolic blood pressure (mean difference: −4.256, 95% CI: −5.99 to −2.52, *P* < 0.0001) in subjects treated with garlic extracts compared to those treated with placebo. Interestingly, trial sequential analysis also supports the observations of the meta-analysis and indicates that a sufficient number of trials have already been performed to reach a consensus conclusion, and further trials are not required. In addition, the GRADEing of evidence also supports the robustness of the observations.

**Conclusions:**

Garlic extracts significantly lower blood pressure and may be prescribed by clinicians for patients with hypertension.

Hypertension, also known as high blood pressure, is a major global health concern affecting people of all ages. According to a recent epidemiological report, it is the leading cause of morbidity and mortality worldwide among all cardiovascular diseases [1]. The prevalence of hypertension is influenced by various factors such as life expectancy, healthcare improvement, and appropriate treatment [2, 3]. Developed countries have higher rates of hypertension, with approximately 20%–30% of adults being affected. The incidence of hypertension in the eastern Mediterranean and Middle Eastern countries has been reported to range from 10% to 17% [2]. The global prevalence of hypertension is projected to increase to 29.2% by 2025, making it even more urgent to take action to address this major cardiovascular risk factor and reduce its burden on global mortality rates [4].

Hypertension management involves not only reducing blood pressure but also preventing complications and maintaining vascular health [5, 6]. Researchers have studied plant products and their potential benefits for the treatment of hypertension. These products can be extracted or isolated from various plants and have shown promising results in reducing blood pressure in individuals with hypertension and normotensive individuals [7, 8], for example, plant extracts from *Carum copticum*, *Tribulus terrestris*, *Nigella sativa*, *Bidens pilosa*, *Vitex doniana*, *Arctium lappa*, *Echinodorus grandiflorus*, *Elettaria cardamomum*, *Daucus carota*, *Apium graveolens*, *Cassia absus*, *Cinnamomum zeylanicum*, *Theobroma cacao*, *Cassia occidentalis*, and *Coriandrum sativum* [7]. However, there is a contradiction in the efficacy of treatment, and appropriate investigations such as controlled trials are lacking.

There is increasing interest in the use of garlic extracts as a natural treatment for hypertension. Several studies have investigated the effects of garlic extracts on blood pressure in patients with hypertension. Although some studies have shown promising results, others have not found any significant effect. Different forms of garlic extracts, such as Kwai, Allicor, Kyoli, aged garlic extracts, Cardiomax, garlic oil, or Dentou-Ninniku-Ranwo, have been tested for their efficacy in reducing blood pressure [9]. Several meta-analyses have been conducted to determine the effectiveness of garlic extracts in managing hypertension [10,11,12,13,14]. However, none of these studies considered uniform study types. Randomized controlled trials (RCTs) are believed to be the most appropriate method for investigating the efficacy of a product. In this meta-analysis, we aimed to include the data from RCTs that investigated the efficacy of garlic in reducing blood pressure. Furthermore, we wish to conduct a sensitivity analysis to test the robustness of the meta-analysis. Additionally, we employed trial sequential analysis (TSA) to determine whether a sufficient number of clinical trials had been conducted to draw firm conclusions.

## Methods

### Database search

Two researchers autonomously searched on databases, such as PubMed, ScienceDirect, and Scopus, to identify pertinent articles for inclusion in the current meta-analysis. Various keywords, such as garlic extract, hypertension, RCTs, placebo, systolic blood pressure (SBP), and diastolic blood pressure, were used for the identification of suitable articles. The last database search was performed on November 25, 2023.

### Eligible criteria of the study

Before extracting data from eligible articles, it is crucial to establish appropriate inclusion and exclusion criteria to conduct a rigorous meta-analysis. The quality of the meta-analysis was highly dependent on the data analyzed. The inclusion criteria in this study were as follows: (i) only placebo-controlled trials were considered; (ii) all subjects had hypertension; (iii) systolic and diastolic blood pressure (DBP) data must be available; (iv) both pretreatment and posttreatment blood pressure data must be available; and (v) the efficacy of garlic extracts or a derivative must have been tested. Articles that did not meet the criteria were excluded, including (i) noncontrolled placebo trials, (ii) reviews and case studies, (iii) reports that lacked blood pressure data, and (iv) papers published in languages other than English.

### Data extraction

Data from the relevant studies were meticulously extracted by two authors, working independently to ensure accuracy and reliability. After evaluating each research article according to the predefined inclusion and exclusion criteria, we extracted the following information from each article with utmost care: name of the first author, year of publication, total number of patients with hypertension in both garlic-treated and placebo groups, type of garlic, manufacturer name, dosage per day, amount of active ingredients per day, treatment duration, mean and standard deviation (SD) of SBP in both garlic-treated and placebo groups before and after treatment, and mean and SD of DBP in both garlic-treated and placebo groups before and after treatment.

### Risk of bias assessment

The Risk of Bias 2 (RoB 2) tool, developed by the Cochrane Collaboration, serves as a comprehensive systematic framework for evaluating the risk of bias in RCTs. This tool assesses 5 essential domains, including the randomization process, deviations from intended interventions, missing outcome data, outcome measurement, and selection of the reported result. Reviewers can determine the risk of bias for each domain by answering specific signaling questions, classifying it as “Low risk,” “Some concerns,” or “High risk.” By combining these assessments, the overall risk of bias for each study can be determined, enhancing the quality and reliability of systematic reviews and meta-analyses. This method provides a rigorous evaluation of the evidence, ensuring its credibility. The risk of bias in the eligible studies was assessed based on the RoB 2 tools [15].

### Statistical analysis

Comprehensive Meta-Analysis Software v4 (CMA) was used to perform the meta-analysis-related statistics. To ensure the accuracy of the results, both Egger’s regression test and funnel plots were used to investigate publication bias, a factor that could potentially skew findings [16]. Additionally, the heterogeneity among the considered reports was examined using *Q* statistics, probability values, and *I*^2^, which allowed researchers to determine the level of variation among the included studies [17]. Depending on the degree of heterogeneity, either a fixed-effects model (homogenous, *P* > 0.05, *I*^2^ < 50) or a random-effects model (heterogeneous, *P* < 0.05, *I*^2^ > 50) was used for the meta-analysis [18]. A threshold of 0.05 was used for statistical significance. To ensure that the meta-analysis results were robust and reliable, a sensitivity analysis was conducted [19]. Furthermore, TSA was performed to determine whether the included trials were sufficient to draw a definitive conclusion or if additional investigations were required [20]. The GRADEing of the evidence was performed by online GRADEPro GDT software [21].

## Results

### Literature search and screening of eligible studies

**Figure 1.**
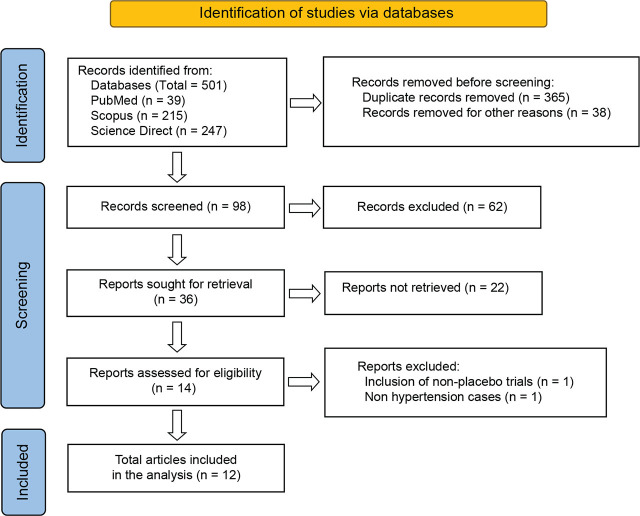
Flow diagram of the studies included in this meta-analysis.

We conducted a search of several databases, including PubMed, Science Direct, and Scopus, to identify potential controlled trials that explored the effectiveness of garlic extracts in treating hypertension. We applied prefixed inclusion and exclusion criteria to select relevant studies and obtained appropriate articles. The details are shown in **Figure 1**, which depicts the PRISMA flowchart. After conducting a database search across PubMed, ScienceDirect, and Scopus, 501 publications were found. After applying the inclusion and exclusion criteria, only 12 studies were included in the meta-analysis [9, 20, 22,23,24,25,26,27,28,29,30,31]. Details of the included reports are shown in **Tables 1 and 2**.

**Table 1.** Characteristics and changes in the SBP of patients with hypertension in garlic-treated cases and controls or placebo

**Author name and year (reference)**	**Population**	**Number of subjects in garlic-treated group/control or placebo group**	**Mean BMI kg/m^2^ (treated/control group)**	**Medication at baseline**	**Manufacturer, dose (mg/d)/duration of treatment (weeks)**	**Mean ± SD SBP pretreated garlic group (mmHg)**	**Mean ± SD SBP post-treated garlic group (mmHg)**	**Mean ± SD SBP pretreated placebo group (mmHg)**	**Mean ± SD SBP post-treated placebo group (mmHg)**
Kandziora [32]	Poland	20/20	NA	Triamterene/hydrochlorothiazide	Kwai, 600/12	178 ± 8	162 ± 9	178 ± 8	173 ± 6
Auer et al. [22]	Germany	24/23	NA	NA	Kwai, 600/12	171 ± 24.5	152 ± 24.5	161 ± 14.4	152 ± 19.2
Vorberg and Schneider [28]	Germany	20/20	NA	NA	Kwai, 900/16	144 ± 10.6	138 ± 4	143.5 ± 10	146 ± 6.5
De Santos and ruenwald [29]	Germany	25/27	NA	NA	Kwai, 900/24	143 ± 21	120 ± 13.1	144 ± 17	145 ± 11.7
Sobenin et al. [26]	Russia	23/19	26.6/27.0	NA	Allicor, 600/12	143.4 ± 7.2	136.8 ± 5.8	140.3 ± 78	139.4 ± 6.5
Sobenin et al. [27]	Russia	64/20	25.4/26.0	NA	Allicor, 2,400/8	154 ± 12.3	147 ± 12.8	149.8 ± 12.6	149.9 ± 11.7
Ried et al. [25]	Australia	8/12	31.0/29.1	ACE inhibitors, A2 receptor antagonists, β-receptor blockers, Ca-antagonists, and diuretics	Kyolic High Potency, 960/12	151.2 ± 7.7	136 ± 8	152.8 ± 9.3	145.4 ± 3.5
Ried et al. [24]	Australia	39/19	29.1/29.9	ACE inhibitors, A2 receptor ant-agonists, β-receptor blockers, Ca-antagonists, diuretics	Kyolic High Potency, 480/12	149.3 ± 13	130 ± 12.8	148.6 ± 13.1	135.9 ± 12.8
Nakasone et al. [23]	Japan	23/24	25.0/23.0	None	Dentou ninniku ranwo, 188/12	141.8 ± 5.6	137 ± 7.8	141.8 ± 5.6	140.4 ± 7.6
Ried et al. [9]	Australia	50/38	27.3/28.3	ACE inhibitors, A2 receptor antagonists, β-receptor blockers, Ca-antagonists, and diuretics	Kyolic Reserve, 1,200/12	148.7 ± 15.3	141.7 ± 15.3	142 ± 9.4	140.3 ± 18.2
Ried et al. [30]	Australia	23/26	28.6/29.7	ACE inhibitors, A2 receptor antagonists, β-receptor blockers, Ca-antagonists, and diuretics	Kyolic Reserve, 1,200/12	153.3 ± 16.4	139 ± 15.1	144.3 ± 14.2	140 ± 16.3
Serrano et al. [31]	Spain	39/38	NA	Thiazide, beta blockers, angiotensin II receptor blocker, and calcium channel blockers	Pharmactive Biotech Spain, 300/12	146 ± 21	143 ± 18	144 ± 13	138 ± 14

NA, data not available; SBP, systolic blood pressure; SD, standard deviation; ACE, angiotensin-converting enzyme; GRADE, Grading of Recommendations Assessment, Development and Evaluation; BMI, body mass index; PRISMA, Preferred Reporting Items for Systematic Reviews and Meta-Analyses.

**Table 2.** Changes in the DBP of patients with hypertension in garlic-treated cases and controls or placebo

**Author name and year**	**Mean ± SD DBP pretreated garlic group (mmHg)**	**Mean ± SD DBP post-treated garlic group (mmHg)**	**Mean ± SD DBP pretreated placebo group (mmHg)**	**Mean ± SD DBP post-treated placebo group (mmHg)**
Kandziora [32]	100 ± 4	85 ± 4	100 ± 3	91 ± 6
Auer et al. [22]	101 ± 14.7	89 ± 4.6	97 ± 9.6	94 ± 9.6
Vorberg and Schneider [28]	91 ± 4	87 ± 4	87.5 ± 6	90 ± 4
De Santos and Gruenwald [29]	89 ± 11	80 ± 4.6	89 ± 11	90 ± 8.3
Sobenin et al. [26]	88.8 ± 4.3	83.8 ± 3.4	87.9 ± 4.8	85.9 ± 4.4
Sobenin et al. [27]	95.7 ± 3.8	92.9 ± 4.4	94.4 ± 6.1	93 ± 6.1
Ried et al. [25]	87.3 ± 7.8	91.9 ± 8.3	88.6 ± 8	84 ± 8.3
Ried et al. [24]	75.7 ± 12.4	68.6 ± 11.6	76 ± 12.2	70.2 ± 12
Nakasone et al. [23]	90.9 ± 6.9	87 ± 7.1	91.6 ± 5.8	90.7 ± 6.5
Ried et al. [9]	89.9 ± 11.7	86.1 ± 11.3	87.8 ± 9.1	86 ± 9
Ried et al. [30]	93 ± 10.9	83.1 ± 10.4	90 ± 12.3	85.5 ± 13
Serrano et al. [31]	85 ± 10	83 ± 9	85 ± 7	83 ± 10

DBP, diastolic blood pressure; SBP, systolic blood pressure; SD, standard deviation.

### Publication bias

**Figure 2.**
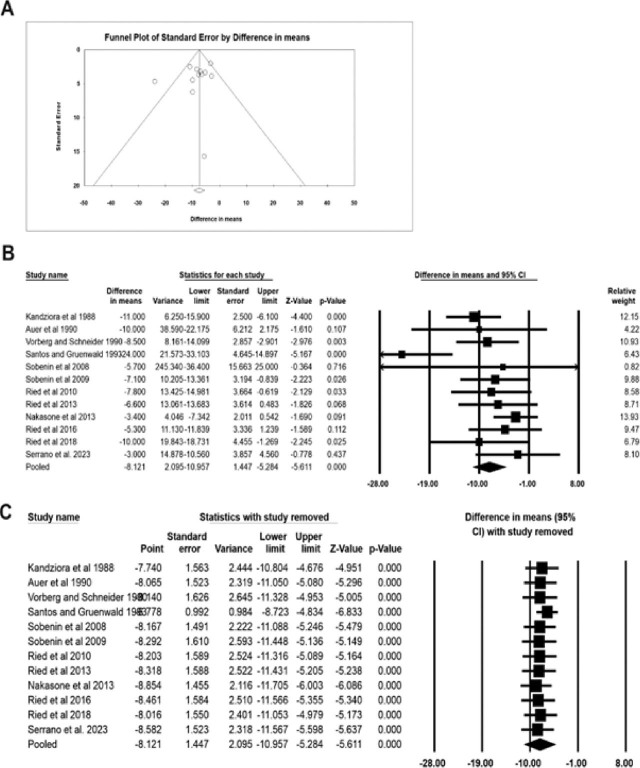
Effect of garlic on diminishing SBP in hypertensive patients. Publication bias in the included reports was explored using funnel plots **(A).** The forest plot revealed a significant decrease in SBP with the treatment of hypertensive patients with garlic compared with those treated with placebo **(B).** The sensitivity analysis supported the robustness of the meta-analysis **(C).** All meta-analysis statistics were performed using CMA v4. CI, confidence interval; CMA, Comprehensive Meta-Analysis; SBP, systolic blood pressure.

**Figure 3.**
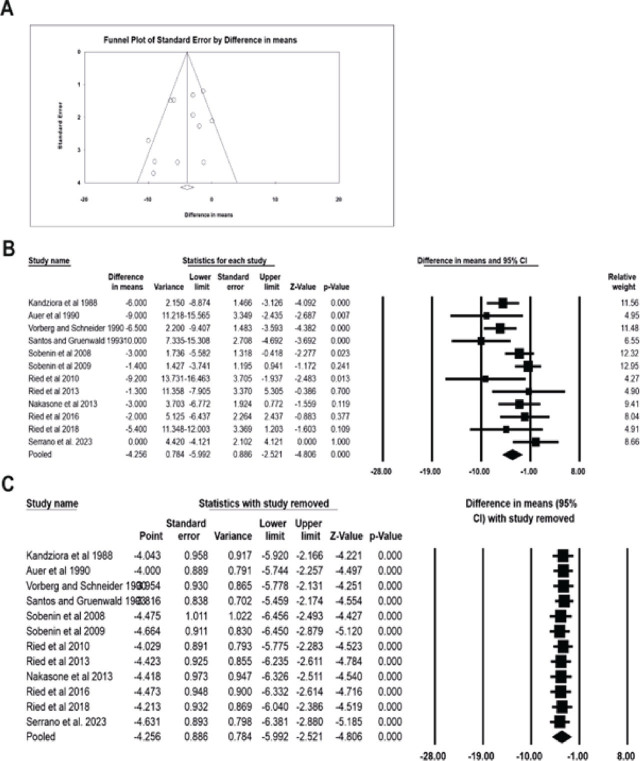
Effect of garlic on diminishing DBP in hypertensive patients. Publication bias in the included reports was explored using funnel plots **(A).** The forest plot revealed a significant decrease in DBP with the treatment of hypertensive patients with garlic compared with those treated with placebo **(B).** The sensitivity analysis supported the robustness of the meta-analysis **(C).** All meta-analysis statistics were performed using CMA v4. CI, confidence interval; CMA, Comprehensive Meta-Analysis; DBP, diastolic blood pressure.

Funnel plots and Egger regression analyses were conducted to examine publication bias. The funnel plot for studying the efficacy of garlic extracts in reducing SBP (**Figure 2A**) and DBP (**Figure 3A**) did not reveal any significant biases. In line with this observation, the Eggers regression analysis indicated the absence of publication bias in the included reports (SBP: intercept = −1.22, 95% confidence interval [CI] = −3.69–1.23, *P* = 0.29; DBP: intercept = −1.38, 95% CI = −3.99–1.21, *P* = 0.26).

### Heterogeneity test

Both the Cochrane *Q* test and *I*^2^ statistics were used to assess the heterogeneity among the reports included in this study. The investigation revealed heterogeneity in SBP (*Q* = 21.22, *P* = 0.03, *I*^2^ = 48.17) and DBP (*Q* = 24.56, *P* = 0.01, *I*^2^ = 55.22). As a result, the random-effects model was used for the meta-analysis to calculate the combined difference in means, standard errors, 95% CI, and probability value.

### Garlic extracts significantly reduced SBP and DBP in patients with hypertension

In the present meta-analysis, 12 trials, comprising 405 patients treated with garlic derivatives and 333 subjects receiving placebo, were considered. As shown in **Figure 2B**, with the administration of garlic extracts, the SBP in hypertensive patients was significantly reduced compared to that in the placebo group (difference in mean: −8.121, 95% CI: −10.95 to −5.28, *P* = 0.000). Similarly, DBP was significantly reduced in the treated group compared to that in the placebo group (difference in mean: −4.256, 95% CI: −5.99 to −2.52, *P* = 0.000) (**Figure 3B**).

### Sensitivity analysis

A sensitivity analysis was conducted to ensure the reliability of the meta-analysis. This involved excluding the data from one report each time the meta-analysis was performed and checking for any deviations from the original result. The consistency of the meta-analysis was confirmed by observing minimal variation in both the sensitivity analysis and the original meta-analysis (**Figures 2C and 3C**), which further justifies the robustness of the findings.

### TSA

TSA is a statistical methodology that is employed in meta-analysis to improve the dependability of conclusions. This methodology aims to control random errors and adjust for multiple testing. TSA calculates the necessary sample size to guarantee that the cumulative evidence is dependable, and it also mitigates the risks of Types I and II errors. TSA utilizes monitoring boundaries to decide if conclusions regarding efficacy or futility can be drawn early, which can potentially save resources by indicating when further trials are not required. This approach helps to prevent spurious findings, accounts for study variability, and reduces the likelihood of biased results, thereby enhancing the overall validity of the conclusions that are reached in meta-analytical studies [20, 33, 34].

**Figure 4.**
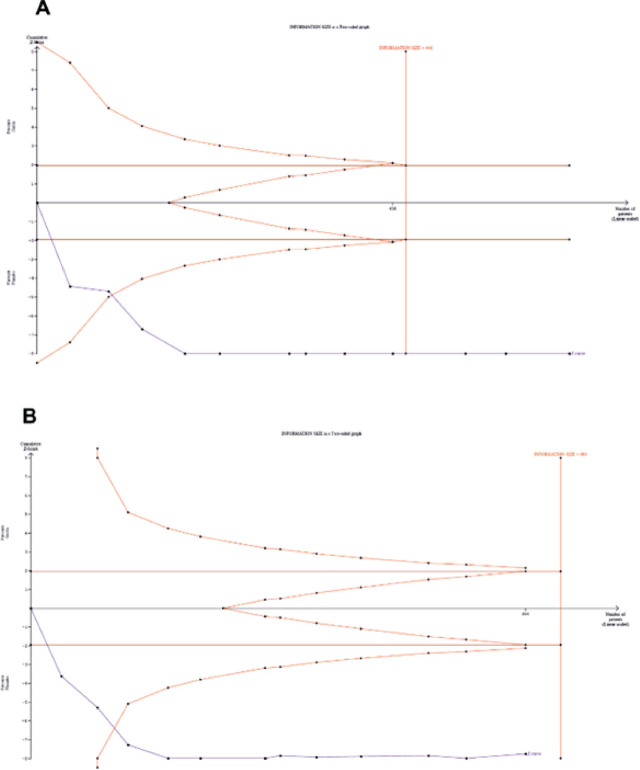
TSA of the reports was performed to investigate the role of garlic extracts in the treatment of hypertension. Reports are considered to investigate the role of garlic in lowering **(A)** SBP and **(B)** DBP; however, further studies are not required. In both TSA plots, the z-curve crosses the alpha spending boundary and reaches the required information size line. DBP, diastolic blood pressure; SBP, systolic blood pressure; TSA, trial sequential analysis.

The efficacy of garlic in reducing SBP and DBP was investigated in this study, and both TSA figures (**Figures 4A,B**) revealed that a sufficient number of trials with sufficient subjects were included to draw a definitive conclusion on the role of garlic in lowering blood pressure in hypertensive patients. The cumulative z-curve crossed the alpha spending boundary and reached the required information size line in both figures, indicating that a satisfactory number of studies have already been considered.

### GRADEing of the evidence

The GRADE Pro GDT software was utilized to evaluate the strength of the evidence derived from the meta-analysis of RCTs, with the aim of determining the significance of garlic extracts in reducing blood pressure among patients with hypertension.

## Discussion

The results of the present meta-analysis suggest that garlic supplementation can significantly reduce both systolic and DBP in patients with hypertension when compared to those who received a placebo. The findings of this study are considered robust, as they were supported by a sensitivity analysis. The investigation also showed that enough studies were included in the meta-analysis with garlic-treated cases and placebos to support the conclusion that garlic plays a significant role in managing hypertension. This means that further studies are not necessary.

The present meta-analysis provides compelling evidence for the effectiveness of garlic extracts in reducing systolic and DBP in patients with hypertension. The results showed a significant decrease in both systolic and DBP in patients who received garlic extracts compared to those who received a placebo. Despite the lack of complete understanding of the specific mechanisms involved, it is believed that the multifaceted nature of garlic extracts and their involvement in various biochemical processes play a significant role in their ability to lower blood pressure. Garlic extracts have been found to inhibit angiotensin-converting enzyme [35], reduce cholesterol synthesis and absorption [36], promote nitric oxide production [37], exert antithrombotic [38] and antioxidant effects [39], and potentially lower blood pressure. Additionally, the gamma-glutamyl cysteine found in garlic may help reduce blood pressure by inhibiting the angiotensin-converting enzyme in vitro [40].

Several meta-analyses have explored the efficacy of garlic extracts in improving hypertension [10,11,12,13,14]. The present meta-analysis had several advantages over previous meta-analyses. First, a larger number of studies was considered in the current analysis. Second, only controlled trials involving garlic extracts were included in the analysis. Third, additional statistical analyses such as sensitivity analysis and TSA imputation were employed in this study. These unique features support the robustness of the meta-analysis and demonstrate that the included studies were sufficient.

The meta-analysis conducted showed that the garlic extract is an effective treatment for hypertension as it reduces both systolic and DBP. However, this study has some limitations that must be considered. First, the search was limited to 3 databases, meaning that articles from other databases may have been excluded. Second, only articles published in English were considered, which increased the possibility of missing relevant articles published in other languages. Third, there was significant publication bias in both the SBP and DBP analysis groups. Finally, there was apparent heterogeneity in both comparisons owing to different populations, types of garlic extracts, quantity of the active compounds, ethnicities, mean blood pressure levels among the studies, and adoption of differential blood pressure measurement methods.

In summary, garlic extracts have been shown to significantly reduce blood pressure and may be recommended by clinicians for the management of hypertension. Given the abundant research that has investigated the efficacy of garlic in managing hypertension, further studies are unnecessary.
